# Difference in Profiles of the Gut-Derived Tryptophan Metabolite Indole Acetic Acid between Transplanted and Non-Transplanted Patients with Chronic Kidney Disease

**DOI:** 10.3390/ijms21062031

**Published:** 2020-03-16

**Authors:** Sophie Liabeuf, Solène M. Laville, Griet Glorieux, Lynda Cheddani, François Brazier, Dimitri Titeca Beauport, Raymond Vanholder, Gabriel Choukroun, Ziad A. Massy

**Affiliations:** 1 Department of Clinical Pharmacology, Amiens University Medical Center, F-80000 Amiens, France; 2 MP3CV Laboratory, EA7517, Jules Verne University of Picardie, Amiens, F-80000 Amiens, France; brazier.francois@chu-amiens.fr (F.B.); titeca.dimitri@chu-amiens.fr (D.T.B.); choukroun.gabriel@chu-amiens.fr (G.C.); 3 Centre for Research in Epidemiology and Population Health (CESP), INSERM UMRS 1018, Université Paris-Saclay, F-94807 Villejuif, France; solene.laville@inserm.fr (S.M.L.); Lynda.cheddani@gmail.com (L.C.); ziad.massy@aphp.fr (Z.A.M.); 4 Nephrology Division, Ghent University Hospital, 9000 Ghent, Belgium; Griet.Glorieux@UGent.be (G.G.); Raymond.Vanholder@UGent.be (R.V.); 5 Department of Nephrology, Amiens University Medical Center, F-80000 Amiens, France; 6 Div of Nephrology, Ambroise Paré University Hospital, APHP, F-92100 Boulogne Billancourt, Paris, France

**Keywords:** uremic toxins, chronic kidney disease, indole acetic acid, kidney transplantation, patient outcome

## Abstract

Background: Uremic toxins have emerged as potential mediators of morbidity and mortality in patients with chronic kidney disease (CKD). Indole-3-acetic acid (IAA, a tryptophan-derived uremic toxin) might be a useful biomarker in patients with CKD. The objectives of the present study were to (i) describe IAA concentrations in a cohort of non-transplanted patients with CKD and a cohort of transplanted patients with CKD, and (ii) investigate the possible relationship between IAA levels and adverse outcomes in the two cohorts. Methods: Levels of free and total IAA were assayed in the two prospective CKD cohorts (140 non-transplanted patients and 311 transplanted patients). Cox multivariate analyses were used to evaluate the association between IAA levels and outcomes (mortality, cardiovascular events, and graft loss). Results: In the non-transplanted CKD cohort, free and total IAA increased progressively with the CKD stage. In the transplanted CKD cohort, free and total IAA levels were elevated at the time of transplantation but had fallen substantially at one-month post-transplantation. Indole acetic acid concentrations were lower in transplanted patients than non-dialysis non-transplanted patients matched for estimated glomerular filtration rate (eGFR), age, and sex. After adjustment for multiple confounders, the free IAA level predicted overall mortality and cardiovascular events in the non-transplanted CKD cohort (hazard ratio [95% confidence interval]: 2.5 [1.2–5.1] and 2.5 [1.3–4.8], respectively). In the transplanted CKD cohort, however, no associations were found between free or total IAA on one hand, and mortality, CV event, or graft survival on the other. Conclusion: We demonstrated that levels of IAA increase with the CKD stage, and fall substantially, even normalizing, after kidney transplantation. Free IAA appears to be a valuable outcome-associated biomarker in non-transplanted patients, but—at least in our study setting—not in transplanted patients.

## 1. Introduction

Patients with chronic kidney disease (CKD) suffer from several metabolic and enzymatic impairments, which result in high morbidity and mortality rates [1]. As kidney function declines, various compounds accumulate progressively. Many of these solutes affect biologic functions and contribute to uremic syndrome, and they are referred to as uremic toxins [2]. 

The gut microbiota has an important role in the generation of precursors of specific uremic toxins associated with poor outcomes in patients with CKD [3]. Both indoxyl sulfate (IS) and indole-3-acetic acid (IAA) originate in the colon, where tryptophan is metabolized by gut bacteria into indole and IAA. Various reports have shown that the accumulation of these uremic toxins in CKD are associated with a decline in kidney function and adverse outcomes [4,5]. However, there are fewer preclinical and clinical data on IAA [5] than on IS [4,6].

Several explanations for the potential toxicity of tryptophan-derived uremic toxins have been put forward, including the activation of inflammation and coagulation pathways [6,7]. Indeed, tryptophan-derived uremic toxins are agonists of the aryl hydrocarbon receptor (AhR) complex. The accumulation of tryptophan-derived indole uremic toxins in patients with CKD may persistently activate the AhR [8], leading to pro-oxidant, pro-inflammatory, pro-coagulant, and pro-apoptotic effects [5].

Most of the clinical data on tryptophan-derived uremic toxins were collected in predialysis or dialyzed patients with CKD. Few studies have evaluated these toxins in transplanted patients with CKD, and no data on IAA are available [9,10,11].

Hence, the objectives of the present study were to (i) describe IAA concentrations in transplanted vs. non-transplanted patients with CKD, and (ii) investigate the putative relationship between IAA concentrations and adverse outcomes.

## 2. Results

### 2.1. Levels of IAA

**Figure 1:**
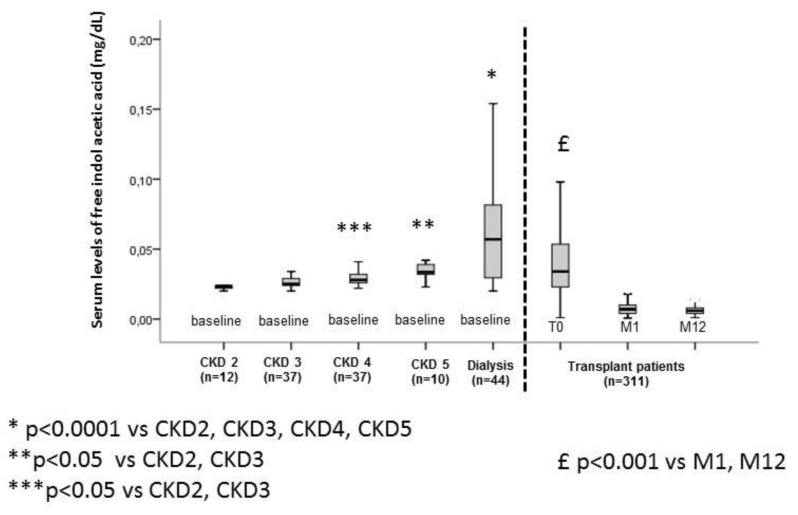
Serum levels of free indole acetic acid in a non-transplanted chronic kidney disease (CKD) cohort and a transplanted cohort.

**Figure 2:**
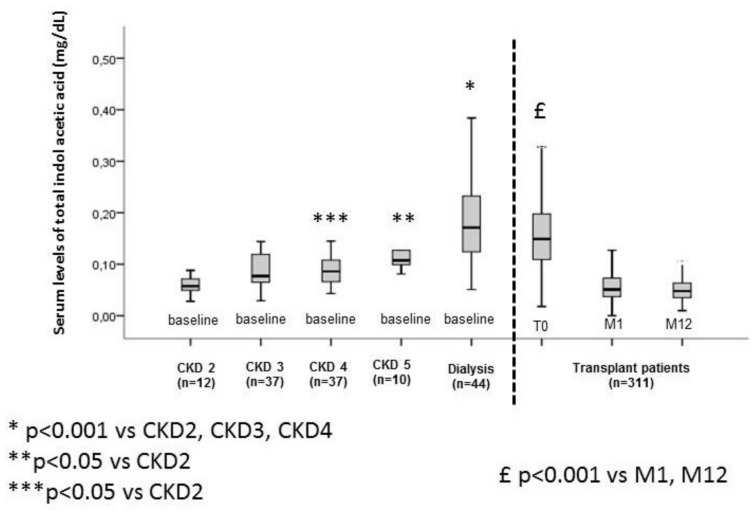
Serum levels of total indole acetic acid in a non-transplanted CKD cohort and a transplanted cohort.

**Figure 3:**
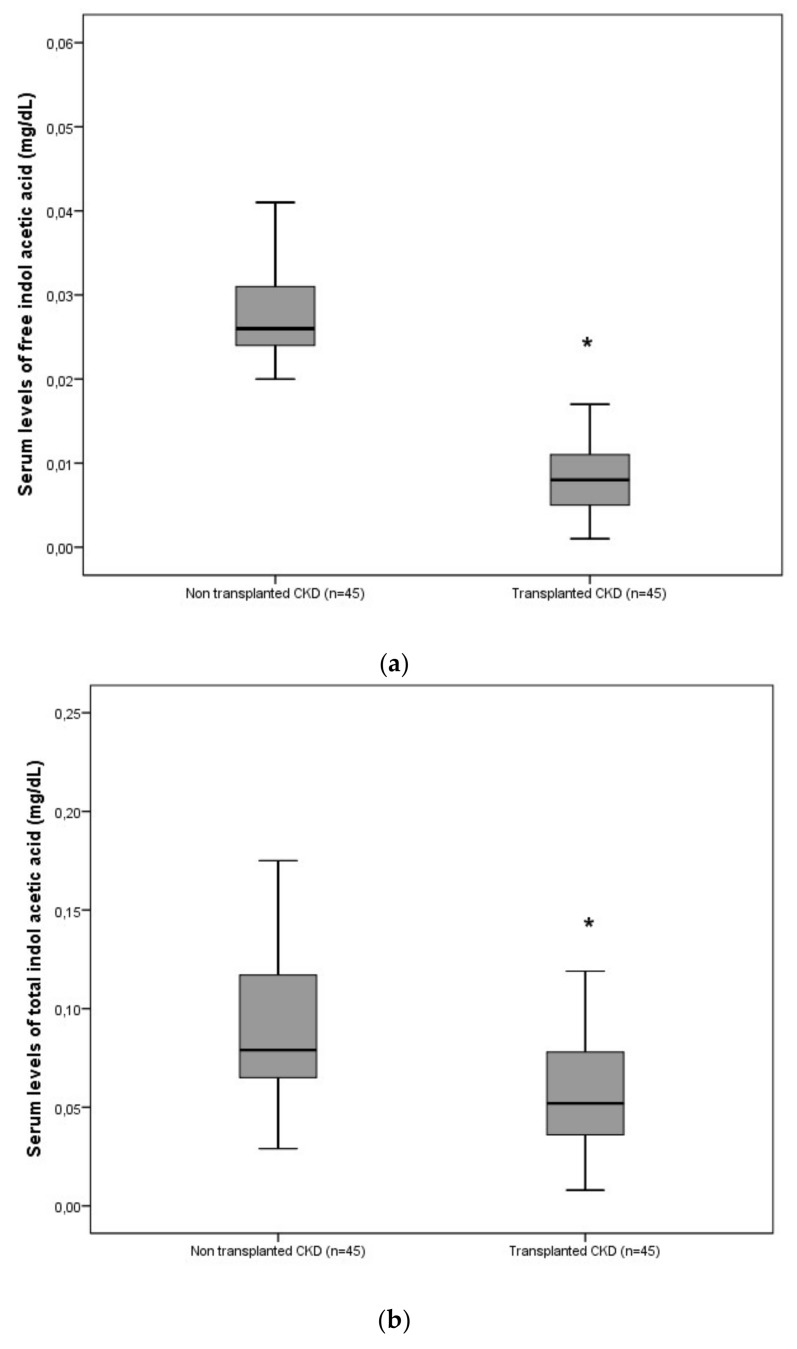
Comparison of mean (range) serum free (**a**) and total (**b**) indole-3-acetic acid (IAA) levels in non-dialized non-transplanted patients with CKD and in transplant recipients at month one (36.0 ± 10.4 mL/mi,/1.73 m^2^) matched for age, sex and estimated glomerular filtration rate (eGFR). (**a**) * *p* < 0.0001; (**b**) * *p* = 0.02. Boxplot with whiskers with maximum 1.5 interquartile range.

The characteristics of the study cohorts are summarized in Table 1. In the cohort of 140 non-transplanted patients with CKD, the free and total IAA increased progressively with the CKD stage, with the highest concentrations in dialyzed patients (Figure 1 and Figure 2). In the cohort of 311 transplanted patients with CKD, the free and total IAA levels were high at the time of transplantation but fell substantially and significantly at one-month post-transplantation and then remained low at 12-months post-transplantation (Figure 1 and Figure 2). After matching 45 patients in the transplanted cohort and 45 non-dialysis patients in the non-transplanted cohort for estimated glomerular filtration rate (eGFR) (mean eGFR = 36 ± 11) and age (mean age = 68 ± 11), we found that mean levels of free and total IAA were significantly lower in transplanted patients (one-month post-transplantation) than in non-transplanted patients (respectively, 0.028 ± 0.007 mg/dL vs. 0.009 ± 0.006 mg/dL for free IAA (*p* < 0.0001) and 0.092 ± 0.042 mg/dL vs. 0.067 ± 0.056 mg/dL for total IAA (*p* = 0.020)) (Figure 3A,B).

**Table 1:** Characteristics of the study cohorts.

Patient Characteristics	Non-Transplanted CKD Cohort (n = 140)	Transplanted Cohort (n = 311)
Age, years	67 ± 12	56 ± 14
Males, %	60	61.8
Diabetes mellitus, %	42	9
Hypertension, %	90	69
Dyslipidemia, %	60	22
History of cardiovascular disease, %	31	23
Active smoker, %	41	17
Body mass index (kg/m^2^)	28 ± 6	25 ± 5
Calcium, mmol/L	2.3 ± 0.2	2.3 ± 0.2
Phosphate, mmol/L	1.3 ± 0.4	0.9 ± 0.5
Proteins, g/L	55.2 ± 6.9	69.0 ± 7.0
Free IAA, mg/dL	0.04 ± 0.03	0.19 ± 0.1
Total IAA, mg/dL	0.12 ± 0.08	2.4 ± 1.1

For the transplanted cohort, the data presented here were collected at the time of transplantation.

Boxplot with whiskers with maximum 1.5 interquartile range.

Boxplot with whiskers with maximum 1.5 interquartile range.

### 2.2. Non-Transplanted Patients with CKD

During the follow-up period (mean duration: 32 ± 12 months), 44 patients died (20 predialysis patients and 24 hemodialyzed patients) and 48 had at least one cardiovascular event. In the study population as a whole, patients with above-median free and total IAA concentrations had a significantly higher risk of overall mortality than patients with below-median levels (*p* = 0.001 for free IAA and *p* = 0.003 for total IAA). Table 2 and Table 3 summarized the results of the Cox regression analyses for the entire population. The free IAA level was still an independent predictor of overall mortality and cardiovascular events after adjustment for multiple confounders (age, dialysis status, CRP, serum albumin, and a history of cardiovascular disease) but total IAA level was not (Table 2).

### 2.3. Transplanted Patients

During the follow-up period (mean duration: 113 ± 29 months), there were 55 deaths, 70 cardiovascular events, and 71 graft losses. Non-adjusted and adjusted models failed to show associations between free and total IAA at the time of transplantation on one hand, and mortality, CV events, or graft survival on the other (Table 3). The same results were obtained when total and free IAA were analyzed 1- and 12-months post-transplantation (data not shown).

## 3. Discussion

In the present study, we measured IAA concentrations, a protein-bound uremic toxin that originates from microbiota metabolism of amino acids and activates the AhR complex, in non-transplanted and transplanted patients with CKD. Our key findings were that (i) in non-transplanted patients, levels of IAA were abnormally high and increased with the CKD stage, (ii) IAA levels fell substantially and significantly within a month of kidney transplantation and remained low after one year, (iii) total and free IAA levels were significantly lower in transplant recipients than in non-recipients matched for age, sex, and eGFR, and (iv) a high free IAA level was an independent risk factor for overall mortality in non-transplanted patients with CKD, whereas free and total IAA levels were not associated with adverse outcomes in patients having received a kidney transplant.

We first observed, in the non-transplanted cohort, that free and total IAA levels changed in a similar way to other protein-bound uremic toxins [4,12,13], i.e., with a progressive increase as the eGFR declined, and the highest levels in dialyzed patients. Next, levels of free and total IAA decreased substantially following kidney transplantation and were lower in kidney transplant recipients than in eGFR-matched patients with CKD. Hence, the change in IAA levels after kidney transplantation was similar to the one seen for other colonic-microbiota-derived uremic retention solutes such as IS, p-cresyl sulfate (PCS), p-cresyl glucuronide, trimethylamine N-oxide, and phenylacetylglutamine [10,11]. There are several possible explanations for the lower levels of uremic toxins measured in kidney transplant recipients, relative to non-transplanted patients with CKD. Firstly, the microbiota might be modified by the transplantation procedure itself or the drugs given after transplantation (such as immunosuppressants and antibiotics) [14]. Indeed, it was previously reported that antimicrobial therapy is likely to alter the composition of the gut microbiota and thus modifies uremic toxin generation [14]. In recent years, many animal and human studies have indicated that gut microbial dysbiosis is closely linked to allogenic transplantation in general (i.e., including kidney transplantation) and post-transplantation complications in particular [15]. Many transplant recipients receive antibiotics and there is a growing body of evidence to show that antibiotic treatment modifies the colonic microbiota and thus microbial metabolism [16].

In recent years, it has been increasingly suspected that the metabolism of the colonic microbiota contributes to the accumulation of uremic retention solutes in CKD (especially IS and PCS). Although both solutes have been linked to adverse outcomes in patients with kidney dysfunction [4,12,13], less attention has been paid to IAA [5]. Dou et al. showed in a cohort of 120 patients at different CKD stages that mortality and cardiovascular events were significantly more frequent in patients with higher levels of IAA [5]. We confirmed these results in a similar cohort. However, we failed to show an association between adverse outcomes in transplanted patients on one hand and IAA levels (regardless of the sample time post-transplantation) on the other.

There are several possible explanations for the positive, independent association between free IAA levels and adverse outcomes in non-transplanted patients with CKD. As for IS, these effects might primarily be related to the activation of the AhR, which appears to have a central role in the biological action of several indoles [17]. High levels of IAA might constitutively activate the AhR and thus lead to pro-oxidant, pro-inflammatory, pro-coagulant, and/or pro-apoptotic effects on the cardiovascular system [17,18].

In patients with CKD who received a kidney transplant, it appears that post-transplantation adverse events were influenced by factors other than the level of IAA—as we previously observed for IS. This finding could be related to the normalization of IAA levels. In addition, in comparison to non-transplanted patients with CKD, kidney transplanted patients presented an increased risk of malignancy-related death and of infectious death that is not likely linked to uremic toxins.

The present study’s main strengths are the assessment of levels of IAA in two CKD cohorts, and the analysis of hard outcomes. In contrast, the main study limitation was the single-center design that nevertheless enabled us to standardize the assays and patient care. Furthermore, a lack of power prevented us from evaluating the impact of IAA on the progression of CKD in the non-transplanted cohort.

In conclusion, our results confirmed that levels of IAA increased in parallel with CKD progression and that kidney transplantation led to a substantial decrease and even normalization in these levels. Free IAA appears to be a valuable biomarker in non-transplanted patients but—at least in our study setting—not in transplanted patients.

## 4. Methods

### 4.1. Study Population

Patients with CKD were recruited into two previously described observational cohort studies [10,19] at Amiens University Medical Center (Amiens, France). All the patients gave their written, informed consent for participation. Both study protocols were approved by the local investigational review board ([PHRC local number: 2006/ 0100 (27/03/2006)], Comité de Protection des Personnes Nord-Ouest II, Amiens, France).

### 4.2. The Transplanted Cohort

Over a 7-year period (from 1 January 1998 to 31 December 2005), a total of 311 patients had a kidney transplantation in the Department of Nephrology, Dialysis and Transplantation at Amiens University Medical Center, and were included in the TOXTRANSLANT study. The included patients consented to blood sample collection at the various study time points: before kidney transplantation (T0) and 1 and 12 months afterwards (M1 and M12). At each study visit, demographic, clinical, laboratory, and treatment-related data were recorded on the basis of the patients’ medical charts. Previous cardiovascular disease was defined as a history of any of the following events: myocardial infarction, stroke, heart failure, angina pectoris, surgical procedures for angina, or coronary/peripheral artery disease (including percutaneous transluminal angioplasty). For descriptive purposes, patients who reported active or historical use of insulin and/or orally administered hypoglycemic drugs were considered to be diabetic. The patients were followed prospectively for death, CV outcomes, and graft loss until 30 September 2011. For deaths, the patient’s medical records were reviewed by a physician and the cause of death was assigned on the basis of all the available clinical information. For out-of-hospital deaths, the patient’s primary care physician was interviewed for relevant information. Cardiovascular events were defined as cardiovascular death or any adverse event directly related to a cardiovascular system dysfunction (stroke, angina pectoris/myocardial infarction, congestive cardiac failure, peripheral ischemia, or new-onset arrhythmia) or surgical procedures for angina or coronary/peripheral arterial disease. Graft loss in transplant recipients was defined as the need for dialysis treatment (because of end-stage kidney disease) for at least 3 consecutive months.

### 4.3. CKD Cohort

Over an 18-month period (from January 2006 to June 2007), a total of 150 Caucasian prevalent patients with CKD were recruited from the Nephrology Department’s outpatient clinic at Amiens University Medical Center. Included patients had to be over the age of 40 with a confirmed diagnosis of CKD. Stage 5D patients with CKD must have received thrice-weekly hemodialysis for at least 3 months. The main exclusion criteria were the presence of chronic inflammatory disease, atrial fibrillation, complete heart block, abdominal aorta aneurysm, aortic and/or femoral artery prosthesis, primary hyperparathyroidism, kidney transplantation, and any acute cardiovascular event in the 3 months before screening for inclusion. The 140 patients who met all the inclusion criteria and had available serum IAA data were included in the analyses.

All patients were day-hospitalized for collection of a blood sample and an interview. Hemodialyzed patients were seen on a dialysis-free day or the morning before their dialysis session, if possible. The patient interview focused on comorbidities and the personal medical history. The patients’ medical charts were reviewed, in order to identify and record any concomitant medications. For descriptive purposes, patients who reported current or previous use of insulin and/or orally administered hypoglycemic drugs were considered to be diabetic. Previous cardiovascular disease was defined as a history of any of the following events: myocardial infarction, stroke, heart failure, angina pectoris, surgical procedures for angina, or coronary/peripheral artery disease (including percutaneous transluminal angioplasty). Cardiovascular events were defined as cardiovascular death or any adverse event directly related to a cardiovascular system dysfunction (stroke, angina pectoris/myocardial infarction, congestive cardiac failure, peripheral ischemia, or new-onset arrhythmia) or surgical procedures for angina or coronary/peripheral arterial disease. Death records were established prospectively. Each medical chart was reviewed, and the cause of death was assigned by a physician on the basis of all the available clinical information. For out-of-hospital deaths, the patient’s family doctor was interviewed to obtain pertinent information on the cause.

### 4.4. Laboratory Tests

In the non-transplanted cohort, blood samples were collected on the day of inclusion only. In the transplanted cohort, blood samples were collected at three time points: immediately before kidney transplantation (T0) and then 1- and 12-months afterwards (M1 and M12). An aliquot of each blood sample was analyzed immediately in an on-site biochemistry and bone biology laboratory, and the remainder was frozen and stored at −80 °C for subsequent quantification of IAA. Serum calcium, phosphate, creatinine, and protein levels were assayed using standard auto-analyzer techniques (the Modular system from Roche Diagnostics, Basel, Switzerland). Free and total levels of IAA in samples from both transplanted and non-transplanted cohorts were measured by reverse-phase high-performance liquid chromatography (HPLC) at the Nephrology Section of the Ghent University Hospital (Ghent, Belgium). The frozen serum samples were thawed, deproteinized by heat denaturation, and then IAA concentrations were quantified using HPLC and fluorescence detection, as described previously [20]. Reference values for healthy subjects were 0.04 ± 0.02 mg/dL and 0.005 ± 0.002 mg/dL for total IAA and free IAA, respectively.

### 4.5. Statistical Analysis

Data were expressed as the mean ± SD or the number (percentage), as appropriate. Levels of IAA were compared using independent- or paired-sample tests, as appropriate. In the two cohorts, univariate and multivariate analyses were performed using Cox proportional hazard models of different outcomes. In the non-transplanted CKD cohort, the outcome was overall mortality. In the transplanted cohort, the outcomes were overall mortality, CV events, and graft loss. For multivariate models, adjustment variables were preselected (on the basis of a literature review) and analyzed in a crude model. Variables with *p* > 0.10 in the crude model were excluded from the multivariate analyses. Various multivariate models were applied for each outcome; the number of adjustment variables in each model was limited by the outcome frequency. In all tests, the threshold for statistical significance was set to *p* ≤ 0.05. Statistical analyses were performed using SPSS software (SPSS Inc., Chicago, IL, USA), version 18.0 for Windows (Microsoft Corp, Redmond, WA, USA).

## Figures and Tables

**Table 2:** A Cox proportional hazards analysis of plasma free and total IAA levels (stratified by the median) for predicting (A) all-cause mortality and (B) cardiovascular events in the non-transplanted CKD cohort (n = 140).

A				
Models	Free IAA		Total IAA	
	HR [95%CI]	*p*	HR [95%CI]	*p*
Unadjusted	3.1 [1.6–6.2]	0.001	2.7 [1.4–5.2]	0.003
+age	3.4 [1.7–6.8]	<0.0001	2.9 [1.5–5.5]	0.001
+age + CRP + hemoglobin	2.3 [1.1–4.9]	0.027	2.9 [1.5–5.5]	0.002
+age + CRP + albumin	2.3 [1.1–4.9]	0.027	2.5 [1.3–4.7]	0.007
+age+ CRP + dialysis	2.5 [1.2–5.1]	0.017	2.0 [0.9–4.1]	0.070
**B**				
**Models**	**Free IAA**		**Total IAA**	
	HR [95%CI]	*p*	HR [95%CI]	*p*
Unadjusted	2.3 [1.3–4.3]	0.006	1.3 [0.7–2.3]	0.378
+age	2.5 [1.4–4.7]	0.002	1.3 [0.8–2.4]	0.322
+age + CRP + hemoglobin	2.5 [1.3–4.6]	0.031	1.3 [0.7–2.3]	0.298
+age + CRP + albumin	2.1 [1.1–3.9]	0.016	1.1 [0.6–2.1]	0.612
+age + CRP+ history of cardiovascular disease	2.5 [1.3–4.6]	0.004	1.3 [0.7–2.3]	0.365
+age + CRP +dialysis	2.5 [1.3–4.8]	0.004	1.1 [0.6–2.2]	0.639

HR = hazard ratio summarizing the effect of above- and below-median levels of IAA (i.e., free IAA > 0.0285 mg/dl vs. with ≤0.0285 mg/dl, and total IAA > 0.101 mg/dl vs. ≤0.101 mg/dl) on the overall mortality risk in an unadjusted model and in models adjusted for the covariates mentioned.

**Table 3:** A Cox proportional hazards analysis of plasma free and total IAA levels at the time of transplantation time (stratified by the median) in predicting (A) all-cause mortality, (B) cardiovascular events and (C) graft loss in the transplanted cohort (n = 311).

A				
**Models**	**Free IAA (T0)**		**Total IAA (T0)**	
	HR [95%CI]	*p*	HR [95%CI]	*p*
Unadjusted	1.0 [0.6–1.8]	0.980	0.8 [0.5–1.5]	0.841
+age	0.8 [0.4–1.4]	0.394	0.9 [0.5–1.6]	0.698
+age + history of cardiovascular disease	1.2 [0.6–2.4]	0.629	1.3 [0.6–2.5]	0.511
+age + history of cardiovascular disease + years on dialysis	1.1 [0.6–2.3]	0.700	1.2 [0.6–2.4]	0.587
**B**				
**Models**	**Free IAA (T0)**		**Total IAA (T0)**	
	HR [95%CI]	*p*	HR [95%CI]	*p*
Unadjusted	1.4 [0.8–2.5]	0.185	1.4 [0.8–2.4]	0.244
+age	1.2 [0.7–2.2]	0.412	1.5 [0.8–2.5]	0.167
+age + history of cardiovascular disease	1.2 [0.6–2.3]	0.634	1.2 [0.6–2.3]	0.610
+age + history of cardiovascular disease + years on dialysis	1.2 [0.6–2.4]	0.517	1.3 [0.7–2.5]	0.478
**C**				
**Models**	**Free IAA (T0)**		**Total IAA (T0)**	
	HR [95%CI]	*p*	HR [95%CI]	*p*
Unadjusted	1.2 [0.7–2.0]	0.569	0.9 [0.5–1.5]	0.570
+age	1.3 [0.7–2.2]	0.378	0.9 [0.5–1.5]	0.609
+age +years on dialysis	1.3 [0.8–2.2]	0.363	1.3 [0.5–1.5]	0.621

H R = hazard ratio summarizing the effect of above- and below-median levels of IAA (i.e., free IAA >0.034 mg/dl vs. ≤0.034 mg/dl, and total IAA >0.149 mg/dl vs. ≤0.149 mg/dl) on the overall mortality risk in an unadjusted model and in models adjusted for the covariates mentioned.
